# Association of ventricular dyssynchrony and strain with cardiac function in patients with repaired tetralogy of Fallot

**DOI:** 10.1186/1532-429X-18-S1-O32

**Published:** 2016-01-27

**Authors:** Linyuan Jing, Jonathan D Suever, Richard Charnigo, Sudad Al hadad, Evan Stearns, Dimitri Mojsejenko, Christopher M Haggerty, Kelsey Hickey, Anne Marie Valente, Tal Geva, Andrew J Powell, Brandon K Fornwalt

**Affiliations:** 1 grid.280776.c0000000403941447Institute for Advanced Application, Geisinger Health System, Danville, PA USA; 2 grid.266539.d0000000419368438Department of Biostatistics, University of Kentucky, Lexington, KY USA; 3 grid.266539.d0000000419368438Saha Cardiovascular Research Center, University of Kentucky, Lexington, KY USA; 4 grid.2515.30000000403788438Department of Cardiology, Boston Children's Hospital, Boston, MA USA

## Background

Patients with repaired tetralogy of Fallot (rTOF) have impairments in cardiac mechanics (strain and dyssynchrony). However, the relationship between cardiac strain, dyssynchrony, and cardiac function (measured by ejection fraction) in patients with rTOF has not been explored comprehensively in a large population. We hypothesized that measures of cardiac mechanics are associated with cardiac function in patients with rTOF.

## Methods

A database search identified patients with rTOF at a single institution who underwent cardiac MRI from May 2005 to March 2012. Left and right ventricular (LV and RV) ejection fraction (EF) were quantified from the MRI and used as measures of cardiac function. Seven parameters of cardiac mechanics were computed using a custom feature-tracking algorithm: LV, RV and inter-ventricular dyssynchrony, as well as LV and RV peak circumferential and longitudinal strains. Dyssynchrony measures and peak circumferential strains were quantified from a stack of short-axis images covering both ventricles, and peak longitudinal strains were quantified from a long-axis four-chamber view. All measures of dyssynchrony were reported as positive numbers, with larger values representing more dyssynchrony. Peak circumferential and longitudinal strains were reported as absolute percentage. A linear regression model was used to determine the correlation between measures of cardiac mechanics and ventricular function.

## Results

153 patients with rTOF (23 ± 14 years, 50% male) were included. LV circumferential strain was strongly correlated with LV EF (r = 0.73, p < 0.001) and moderately with RV EF (r = 0.43, p < 0.001) (Figure). Similarly, RV circumferential strain correlated with RV EF (r = 0.70, p < 0.001) and LV EF (r = 0.46, p < 0.001) (Figure). Both LV and RV longitudinal strains were moderately correlated with LV and RV EF (Table 1). Intra-ventricular (both LV and RV) dyssynchrony was weakly correlated with LV and RV EF (all |r| ≤ 0.26) (Table). Inter-ventricular dyssynchrony was modestly correlated with RV EF (r = -0.41, p < 0.001), but not with LV EF (r = -0.04, p = 0.58).

**Table 1:** Summary of associations between cardiac dyssynchrony/strain and ventricular function

	RVEF (%)		LV EF (%)	
	r	p	r	p
LV dyssynchrony (ms)	-0.17	0.03	-0.26	<0.001
RV dyssynchrony (ms)	-0.20	0.01	-0.21	0.008
Inter-V dyssynchrony (ms)	-0.41	<0.001	-0.04	0.58
LV circ. strain (%)	0.43	<0.001	0.73	<0.001
RV circ. strain (%)	0.70	<0.001	0.46	<0.001
LV long. strain (%)	0.46	<0.001	0.47	<0.001
RV long. strain (%)	0.46	<0.001	0.35	<0.001

**Figure 1:**
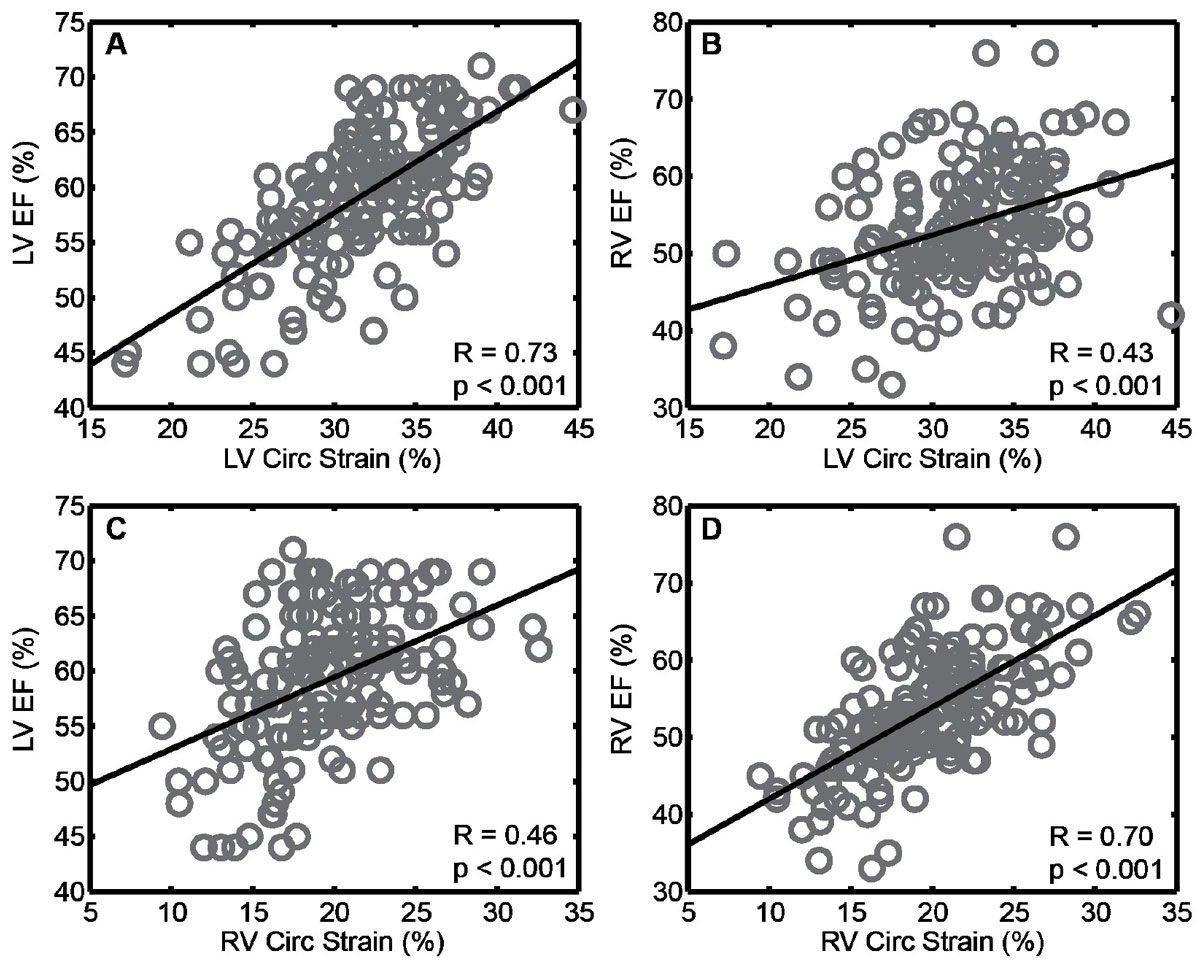
**Correlation between peak circumferential strain and ejection fraction**. A: LV circumferential strain vs LV EF; B: LV circumferential strain vs RV EF; C: RV circumferential strain vs LV EF; D: RV circumferential strain vs RV EF.

## Conclusions

In patients with rTOF, measures of ventricular dyssynchrony and strain are associated with ventricular function measured by ejection fraction. The correlations between LV mechanics and RV function (and vice versa) may indicate unfavorable ventricular-ventricular interaction. The weak association between ventricular dyssynchrony and cardiac function suggests that dyssynchrony and dysfunction may occur independently in patients with rTOF.

